# Can Epstein–Barr virus‐deoxyribonucleic acid load after induction chemotherapy combined with American Joint Committee on Cancer stage determine the chemotherapy intensity of locally advanced nasopharyngeal carcinoma?

**DOI:** 10.1002/cam4.4899

**Published:** 2022-06-08

**Authors:** Qun Zhang, Zhen‐Wei Peng, Zhuo‐Sheng Gu, Yan Wang, Fang He, Wen‐Bin Zhao, Wei Luo, Yong‐Yu Mei

**Affiliations:** ^1^ Department of Radiotherapy First Affiliated Hospital of Sun Yat‐sen University Guangzhou People's Republic of China; ^2^ Department of Radiotherapy Sun Yat‐sen University Cancer Center, State Key Laboratory of Oncology in South China, Collaborative Innovation Center for Cancer Medicine Guangzhou People's Republic of China; ^3^ Department of Radiotherapy Sixth Affiliated Hospital of Sun Yat‐sen University Guangzhou People's Republic of China; ^4^ Department of Infectious Diseases Third Affiliated Hospital of Sun Yat‐sen University Guangzhou People's Republic of China

**Keywords:** chemotherapy intensity, Epstein–Barr virus DNA, induction chemotherapy, locoregionally advanced, nasopharyngeal carcinoma

## Abstract

**Background:**

Induction chemotherapy (IC) comprising docetaxel, cisplatin, and fluorouracil (TPF), combined with concurrent chemoradiotherapy (CCRT) effectively improves the survival rate of locally advanced nasopharyngeal carcinoma (LA‐NPC). Selecting patients whose risk of tumor recurrence and metastasis is high and the appropriate chemotherapy intensity is a concern. We combined tumor‐node‐metastasis staging with the load of Epstein–Barr virus (EBV) after IC to select the individualized chemotherapy strength.

**Methods:**

The clinical data and prognostic factors of patients with stage III–IV LA‐NPC treated with TPF IC combined with CCRT were analyzed retrospectively. The conventional treatment group received the standard three cycles TPF IC combined with CCRT. For the new treatment group, the cycles of IC were determined according to whether the EBV‐DNA disappeared completely after a certain course of IC, if so, subsequent IC was stopped and the chemoradiotherapy stage was entered. Propensity score matching (PSM) was performed at a ratio of 1:1 to balance baseline characteristics. Survival outcomes and adverse events between the conventional treatment group and the new method treatment group were compared.

**Results:**

The study included 256 patients, among whom 192 were matched successfully into 96 pairs. The patients were followed up for a median of 51 months. The proportions of patients receiving three, two, and one cycle of IC after PSM in the routine and new treatment cohorts were 93.8%, 3.1%, 3.1% versus 21.9%, 49.0%, 24.0%, respectively. However, their 3‐year distant metastasis‐free survival, local recurrence‐free survival, progression‐free survival, and overall survival did not differ significantly. The incidence of grade 3–4 neutropenia toxicity in CCRT decreased significantly in patients receiving the new treatment method compared with that in the conventional treatment group (*p* = 0.026).

**Conclusion:**

Combining TNM stage and EBV‐DNA load after IC to determine the courses of IC in patients with LA‐NPC did not alter the curative effect but decreased toxicity.

## Graphical abstract

The cycles of induction chemotherapy IC were determined according to whether the EBV‐DNA disappeared completely after a certain course of IC. In the new method group, the cycles of induction chemotherapy were reduced by 28%, and the serious neutropenia toxicity was decreased by 10% during concurrent chemoradiotherapy, comparted with the routine strategy. However, their 3‐year distant metastasis free survival, local recurrence free survival, progression free survival, and overall survival did not differ significantly.

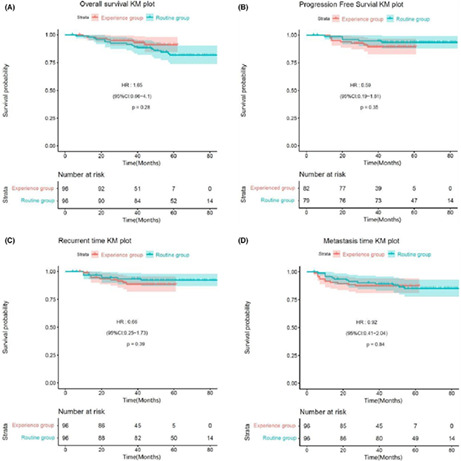

## 1 INTRODUCTION

In Southeast China, nasopharyngeal cancer (NPC) is endemic, affecting about 20–50 per 100,000 people.^1^, ^2^, ^3^ Treatment using concomitant chemotherapy (CT) and intensity‐modulated radiotherapy (IMRT) has decreased the frequency of locoregional relapse; however, distant recurrence is still a major problem.^4^, ^5^ Currently, the National Comprehensive Cancer Network (NCCN) recommendations comprise concurrent CT plus adjuvant CT or induction CT (IC), followed by concurrent chemoradiotherapy (CCRT) as level 2A evidence for local advanced nasopharyngeal carcinoma.^6^ However, treatment with IC or adjuvant CT alone has no significant benefit.^7^, ^8^, ^9^, ^10^ Patients are more tolerant of IC better, and IC is better at eradicating micrometastases earlier, compared with adjuvant sequencing. As a result, IC followed CCRT might be a promising method to treat NPC in the era of IMRT.^11^, ^12^, ^13^ However, the results of randomized trials regarding the efficacy of additional IC are inconsistent. Moreover, there is no established ideal IC intensity.^14^, ^15^, ^16^, ^17^ Furthermore, the additional benefit of IC to some patients with low risk is controversial. Our previous study found that the prolongation of the total treatment time was an independent adverse factor affecting the therapeutic effect.^18^ However, at the moment, treatment delivery and risk stratification refer mainly to the tumor‐node‐metastasis (TNM) staging, which might not be sufficient to identify patients at low risk.^19^, ^20^ Therefore, powerful factors to aid treatment strategy selection and risk stratification should be identified urgently.

In NPC screening, Epstein–Barr virus (EBV) DNA levels in plasma are an independent prognostic biomarker for NPC, with a sensitivity of 97.1% and a specificity of 98.6%.^21^, ^22^ In addition, a poor EBV DNA response after IC, mid‐course of radiotherapy, or post‐treatment, indicated an adverse prognosis for clinical outcome.^23^, ^24^, ^25^ Jiawei Lv found a substantial proportion of complete responders (approximately 30%) in terms of cell‐free (cf) EBV‐DNA after only one IC cycle.^26^ Therefore, this study aimed to investigate the utility of liquid biopsy of the response of EBV‐DNA as a biomarker to adapt risk‐stratified treatment.

## 2 MATERIALS AND METHODS

### 2.1 Study design and participants

For this trial, we recruited patients with a new diagnosis of stage III–IVA NPC treated from July 2011 to April 2019. The patients received induction chemotherapy (IC) regimens of docetaxel, cisplatin, and fluorouracil (TPF), plus two cycles cisplatin CCT from an attending doctor's team at Sun Yat‐sen University Cancer Center. Pretreatment, cfEBV‐DNA was detectable in the patients. Patients were recruited based on the following inclusion criteria: Age > 18 years old diagnosed histologically with LA‐NPC at the Union for International Cancer Control (UICC; 8th edition) stage III or IVA; an Eastern Collaborative Oncology Group performance status of 0 to 1; sufficient bone marrow and organ function; and pretreatment EBV DNA >0 copies/ml.

The patients were treated with two treatment schemes: The conventional treatment group was treated with standard three‐cycle TPF IC combined with CCRT. In the new treatment group, the cycles of IC were further determined according to whether the EBV‐DNA disappeared completely after a certain course of IC. Once the post‐IC EBV‐DNA dropped to 0, the subsequent IC was stopped and the chemoradiotherapy phase was entered.

Propensity score matching (PSM) at a ratio of 1:1 was performed to balance the baseline characteristics. For the two groups, survival and adverse events were compared.

### 2.2 Clinical staging

The patients received a pretreatment physical examination. The patients were then subjected to imaging, including tomography or radiography of the chest, computed tomography or sonography of the abdomen, a whole‐body bone scan, and nasopharynx and neck magnetic resonance imaging (MRI). If indicated clinically, positron emission tomography‐computed tomography (PET‐CT) was carried out. Staging of the patients was carried out according to the International Union against Cancer/American Joint Committee on Cancer (UICC/AJCC) staging system manual (8th edition).

### 2.3 Quantitative real‐time PCR assay for EBV‐DNA

Quantitative real‐time polymerase chain reaction (qPCR) was carried out to measure the pretreatment level of EBV‐DNAs. The qPCR system initially detected the *Bam* HI‐W region. cfEBV‐DNA analysis was performed at various time points: Pretreatment (2 weeks before IC initiation), after each cycle of IC (post‐IC), and within and during 1 week of CCRT completion (post‐CCRT).

### 2.4 Chemotherapy procedure

The patients were treated using two treatment schemes: The conventional treatment group was treated with standard three‐cycle TPF IC plus CCRT. In the new treatment group, once the post‐ICT EBV‐DNA load dropped to 0, these patient did not receive further IC and were treated with CCRT directly. The administration of IC was carried out as follows: docetaxel at 60 mg/m^2^ on day 1, cisplatin at 65 mg/m^2^ on day 1, and 5‐ fluorouracil at 550 mg/m^2^ on days 1–5, repeated every 3 weeks. Concurrent CT comprised cisplatin at 80 mg/m^2^ on day 1, repeated every 3 weeks. The chemotherapy dose setting was based on the results of our previously published dose climbing trial from 2009 to 2010.^27^ According to the protocol, when a patient has experienced severe toxicity, such as: neutrophils <0.5 × 10^9^/L, agranulocytosis fever, granulocyte deficiency infection, platelets <25 × 10^9^/L, alanine aminotransferase/aspartate aminotransferase > 2.5 to <5 × the upper limit of normal, grade 2 neurotoxicity and nephrotoxicity, grade 3–4 mucositis and diarrhea, for the first time during chemotherapy, the administration was continued after reducing the dose by 20%. If the toxicity lasted for more than 2 weeks, permanent drug withdrawal was considered according to the judgment of the doctor.^18^ Written informed consent was provided by all the patients and the Sun Yat‐Sen University Cancer Center Review Board (Guangzhou, China) approved the experimental protocol.

### 2.5 Radiotherapy process

Radical IMRT was delivered to all patients. The prescribed radiation doses comprised 66–70 Gy to the involved neck area and 70 Gy to the primary tumor. All bilateral cervical lymphatics and potential local infiltration sites were irradiated at 54 Gy or greater. All patients received 33 fractions over 6–7 weeks at five daily fractions per week.

### 2.6 Strategy for follow‐up

Follow‐up of patients using imaging methods was carried out every 3 months in the first 2 years, every 6 months in years 3–5, and yearly thereafter. Follow‐up duration was calculated from first treatment day to the last visit or death. Disease‐free survival (DFS, representing the time to the first event or death from any cause) was the primary endpoint. Overall survival (OS, representing the time to death from any cause), distant metastasis‐free survival (DMFS, representing the time to first distant failure), and locoregional relapse‐free survival (LRRFS, representing the time to first local or regional recurrence or both) comprised the other endpoints. Routine plasma EBV‐DNA determination, radiography of the chest, ultrasound of the abdomen, neck, and nasopharynx contrast‐enhanced MRI, and nasopharyngoscopy were carried out. PET/CT was performed only if considered necessary. Adverse events were graded using The Common Terminology Criteria for Adverse Events (version 5).

The late toxicity of salivary glands, subcutaneous tissue, and skin were assessed using the Radiation Therapy Oncology Group and European Organization for Research and Treatment of Cancer Late Radiation Morbidity Scoring Criteria.

### 2.7 Statistical analysis

R software version 4.0.2^28^ was used to carry out most analyses. The Kaplan–Meier method was used to construct survival curves, which were compared employing the log‐rank test. For multivariate analyses, the Cox proportional hazards model was employed. To balance factors and match patients with the routine treatment group and the new method group, we used PSM at a ratio of 1:1. Matching covariates comprised clinical stage, N stage, T stage, body mass index (BMI), sex, and aged. The categorical variables were compared using Fishers' exact test or a chi‐squared test, and Students *t*‐test was carried out to compare continuous variables.

## 3 RESULTS

### 3.1 Patient and treatment characteristics

Between July 2011 and June 2019, 256 patients with stage III–IV NPC were recruited and gave consent. The conventional treatment group comprised 150 patients and the new treatment group comprised 106 patients. Following PSM analysis, we selected 192 patients (96 pairs). The patients' clinical characteristics were well balanced between the routine and new treatment groups (Table 1).

**TABLE 1:** Characteristics of the whole and propensity score‐matched cohorts of patients with NPC received routine or new treatments

Characteristic	Entire cohort no. (%)^a^				Propensity score‐matched cohorts no. (%)^a^			
	Total	Routine group	New treatment group	*p*	Total	Routine group	New treatment group	*p*
Age, years	256	150 (58.6)	106 (41.4)	0.254	192	96 (50)	96 (50)	0.787
<18	0 (0.0)	0 (0.0)	0 (0.0)		0 (0.0)	0 (0.0)	0 (0.0)	
18–37	71 (27.7)	41 (27.3)	30 (28.3)		50 (26.0)	25 (26.0)	27 (28.1)	
38–45	57 (22.3)	38 (25.3)	19 (17.9)		42 (21.9)	23 (24.0)	17 (17.7)	
46–52	62 (24.2)	38 (25.3)	24 (22.6)		42 (21.9)	21 (21.9)	23 (24.0)	
≥53	66 (25.8)	33 (22.0)	33 (31.1)		58 (30.2)	27 (28.1)	29 (30.2)	
Gender				0.355				1.000
Male	201 (78.5)	121 (80.7)	80 (75.5)		150 (78.1)	74 (77.1)	75 (78.1)	
Female	55 (21.5)	29 (19.3)	26 (24.5)		42 (21.9)	22 (22.9)	21 (21.9)	
UICC/AJCC clinical stage (8th edition)				0.116				1.000
III	96 (37.5)	50 (33.3)	46 (43.4)		83 (43.2)	41 (42.7)	42 (43.8)	
IVa	160 (62.5)	100 (66.7)	60 (56.6)		109 (56.8)	55 (57.3)	54 (56.2)	
T category (8th edition)				0.006				0.744
T1	6 (2.3)	3 (2.0)	3 (2.8)		4 (2.1)	2 (2.1)	2 (2.1)	
T2	32 (12.5)	22 (14.7)	10 (9.4)		16 (8.3)	6 (6.3)	10 (10.4)	
T3	104 (40.6)	48 (32.0)	56 (52.8)		94 (49.0)	47 (49.0)	47 (49.0)	
T4	114 (44.5)	77 (51.3)	37 (34.9)		78 (40.6)	41 (42.7)	37 (38.5)	
N category (8th edition)				0.429				0.851
N0	0 (0.0)	0 (0.0)	0 (0.0)		0 (0.0)	0 (0.0)	0 (0.0)	
N1	55 (21.5)	37 (24.7)	18 (17.0)		37 (19.3)	20 (20.8)	18 (18.8)	
N2	135 (52.7)	77 (51.3)	58 (54.7)		109 (56.8)	54 (56.2)	55 (57.3)	
N3	66 (25.8)	36 (24.0)	30 (28.3)		46 (24.0)	22 (22.9)	24 (25.0)	
BMI (kg/m^2^)^c^				0.936				0.722
<18.5	26 (10.2)	15 (10.1)	11 (10.4)		18 (9.4)	9 (9.4)	9 (9.4)	
≥18.5 < 23.9	138 (54.0)	79 (53.0)	59 (55.7)		100 (52.1)	46 (47.9)	54 (56.2)	
≥ 23.9 < 27	67 (26.2)	42 (28.2)	25 (23.6)		52 (27.1)	30 (31.2)	22 (22.9)	
≥27 < 31	17 (6.6)	9 (6.0)	8 (7.5)		15 (7.8)	7 (7.3)	8 (8.3)	
≥31	8 (3.1)	4 (2.7)	3 (2.8)		7 (3.6)	4 (4.2)	2 (2.1)	
HB (g/L)^c^				0.585				0.770
<110	8 (3.1)	5 (3.4)	3 (2.8)		5 (2.6)	3 (3.1)	2 (2.1)	
≥110 < 150	172 (67.2)	96 (64.0)	75 (70.8)		130 (67.7)	63 (65.6)	67 (69.8)	
≥150	76 (29.7)	48 (32.0)	28 (26.4)		57 (29.7)	30 (31.2)	27 (28.1)	
White blood cell (10 × 10^9^/L)^c^				0.692				1.000
<4	6 (2.3)	4 (2.7)	2 (1.9)		4 (2.1)	2 (2.1)	2 (2.1)	
≥4 < 10	230 (89.8)	135 (90.0)	94 (88.7)		173 (90.1)	87 (90.6)	86 (89.6)	
≥10	20 (7.8)	10 (6.7)	10 (9.4)		15 (7.8)	7 (7.3)	8 (8.3)	1.000
NLR^c^				0.696				
<2.7	156 (60.9)	93 (62.0)	63 (59.4)		119 (62.0)	59 (61.5)	60 (62.5)	
≥2.7	100 (39.1)	56 (37.3)	43 (40.6)		73 (38.0)	37 (38.5)	36 (37.5)	
C‐reactive protein (mg/L)^c^				0.555				0.700
<1	65 (25.4)	38 (25.3)	27 (25.5)		50 (26.0)	25 (26.0)	25 (26.0)	
≥1 < 3	71 (27.7)	38 (25.3)	33 (31.1)		55 (28.6)	25 (26.0)	30 (31.2)	
≥3	120 (46.9)	74 (49.3)	46 (43.4)		87 (45.3)	46 (47.9)	41 (42.7)	
LDH (μ/L)^c^				0.845				0.479
<245	226 (88.3)	133 (88.7)	93 (87.7)		172 (89.6)	88 (91.7)	84 (87.5)	
≥245	30 (11.7)	17 (11.3)	13 (12.3)		20 (10.4)	8 (8.3)	12 (12.5)	
Pre EBV DNA (copies/ml)^c^				0.004				0.023
<1000	47 (18.4)	20 (13.3)	28 (26.4)		39 (20.3)	13 (13.5)	26 (27.1)	
≥1100 < 10,000	133 (52.0)	58 (38.7)	75 (70.8)		101 (52.6)	34 (35.4)	67 (69.8)	
≥10,000	14 (5.5)	11 (7.3)	3 (2.8)		12 (6.3)	9 (9.4)	3 (3.1)	0.560
Concomitant disease^b^				0.602				
Hepatitis	22 (8.6)	12 (8.0)	10 (9.4)		16 (8.3)	7 (7.3)	9 (9.4)	
Diabetes	11 (4.3)	5 (3.3)	6 (5.7)		8 (4.2)	3 (3.1)	5 (5.2)	
Both	2 (0.8)	2 (1.3)	0 (0.0)		2 (1.0)	2 (2.1)	0 (0.0)	
NO	221 (86.3)	131 (87.3)	90 (84.9)		166 (86.5)	84 (87.5)	82 (85.4)	
Total treatment time (day)				<0.001				<0.001
≤115	119 (46.5)	44 (29.3)	75 (70.8)		90 (46.9)	21 (21.9)	69 (71.9)	
>115	137 (53.5)	106 (70.7)	31 (29.2)		102 (53.1)	75 (78.1)	27 (28.1)	
GAP				<0.001				<0.001
≤68 day	125 (48.8)	48 (32.0)	77 (72.6)		94 (49.0)	24 (25.0)	70 (72.9)	
>68 day	131 (51.2)	102 (68.0)	29 (27.4)		98 (51.0)	72 (75.0)	26 (27.1)	
IC cycles				<0.001				<0.001
1	41 (16.0)	13 (8.7)	29 (27.4)		26 (13.5)	3 (3.1)	23 (24.0)	
2	54 (21.1)	7 (4.7)	47 (44.3)		50 (26.0)	3 (3.1)	47 (49.0)	
3	154 (60.2)	130 (86.7)	24 (22.6)		111 (57.8)	90 (93.8)	21 (21.9)	
4	7 (2.7)	0 (0.0)	7 (6.7)		5 (2.6)	0 (0.0)	5 (5.2)	
CCT cycles				0.542				1.000
1	11 (4.3)	5 (3.3)	6 (5.7)		6 (3.1)	3 (3.1)	3 (3.1)	
2	240 (93.8)	142 (94.7)	97 (91.5)		183 (95.3)	92 (95.8)	91 (94.8)	
3	5 (2.0)	2 (1.3)	3 (2.8)		3 (1.6)	1 (1.0)	2 (2.1)	
Response NP				<0.001				<0.001
CR	177 (69.1)	125 (83.3)	52 (49.1)		126 (65.6)	78 (81.2)	48 (50.0)	
PR	78 (30.5)	24 (16.0)	53 (50.0)		65 (33.9)	18 (18.8)	47 (49.0)	
SD	1 (0.4)	0 (0.0)	1 (0.9)		1 (0.0)	0 (0.0)	1 (1.0)	
Response ND				0.069				
CR	182 (71.1)	113 (75.3)	69 (65.1)		136 (70.8)	74 (77.1)	62 (64.6)	0.080
PR	74 (28.9)	36 (24.0)	37 (34.9)		56 (29.2)	22 (22.9)	34 (35.4)	

*Note*: *p*‐values were two‐sided with significance defined as a *p*‐value <0.05.

Abbreviations: AJCC, American joint Committee on Cancer; CCT, concurrent chemotherapy; CR, complete remission; DNA, deoxyribonucleic acid; EBV, Epstein–Barr virus; GAP, RT initiation date—IC initiation date; IC, induction chemotherapy; ND = cervical lymph nodes; NP, nasopharynx; NPC, nasopharyngeal carcinoma; PR, partial remission; SD, stable disease; TTT, all treatment completion date—IC initiation date; UICC, Union for International Cancer Control.

^a^ Percentages may not add up to 100 due to rounding.

^b^ All chemoradiotherapy was based on intensity‐modulated radiation therapy.

^c^ All variables were measured before treatment.

For the whole cohort: The proportions of patients who received three cycles, two cycles, and one cycle of IC in the routine and new treatment cohorts were 86.7%,4.7%, and 8.7% versus 22.6%, 44.3%, and 27.4%, respectively. Following PSM analysis, the proportions of patients who received three cycles, two cycles, and one cycle of IC in the routine and new treatment cohorts were 93.8%, 3.1%, and 3.1% versus 21.9%, 49.0%, and 24.0%, respectively.

Cisplatin was used in combination with radiotherapy. Most of the patients in the cohort were treated with ≥160 mg/m^2^ cumulative concurrent dose (CCD) of cisplatin (*N* = 245 (95.7%); 11 (4.3%) received CCD < 160 mg/m^2^). The baseline characteristics of the patients and the treatment details pre‐ and post‐PSM analysis are shown in Table 1.

### 3.2 Effects of treatment

For the whole and matched cohorts, the median durations of follow‐up were 54 months (range: 4–89 months) and 49 months (range: 6–89 months). In the whole cohort, we recorded 16 (6.3%) locoregional recurrences, 31 (12.1%) distant metastases, 5 (2.0%) synchronous locoregional and metastatic recurrences, and 52 (20.3%) disease progress. In the convention therapy group and new treatment group, 8 (5.3%), 20 (13.3%), 4 (2.7%), and 32 (21.3%) versus 8 (7.5%), 11 (10.4%), 1 (1.0%), and 20 (18.9%) patients, respectively, experienced locoregional recurrence, distant metastasis, synchronous locoregional and metastatic recurrences, and disease progress. In the matched cohort, the proportions of locoregional recurrence, distant metastasis, synchronous locoregional and metastatic recurrence, and disease progress were 5 (5.2%), 9 (9.4%), 3 (3.1%), and 17 (17.7%) in the convention therapy group. In the new treatment group, 8 (8.3%), 10 (10.4%), 1 (1.0%), and 19 (19.8%) patients, respectively, developed locoregional recurrence, distant metastasis, synchronous locoregional and metastatic recurrences, and disease progress.

**FIGURE 1:**
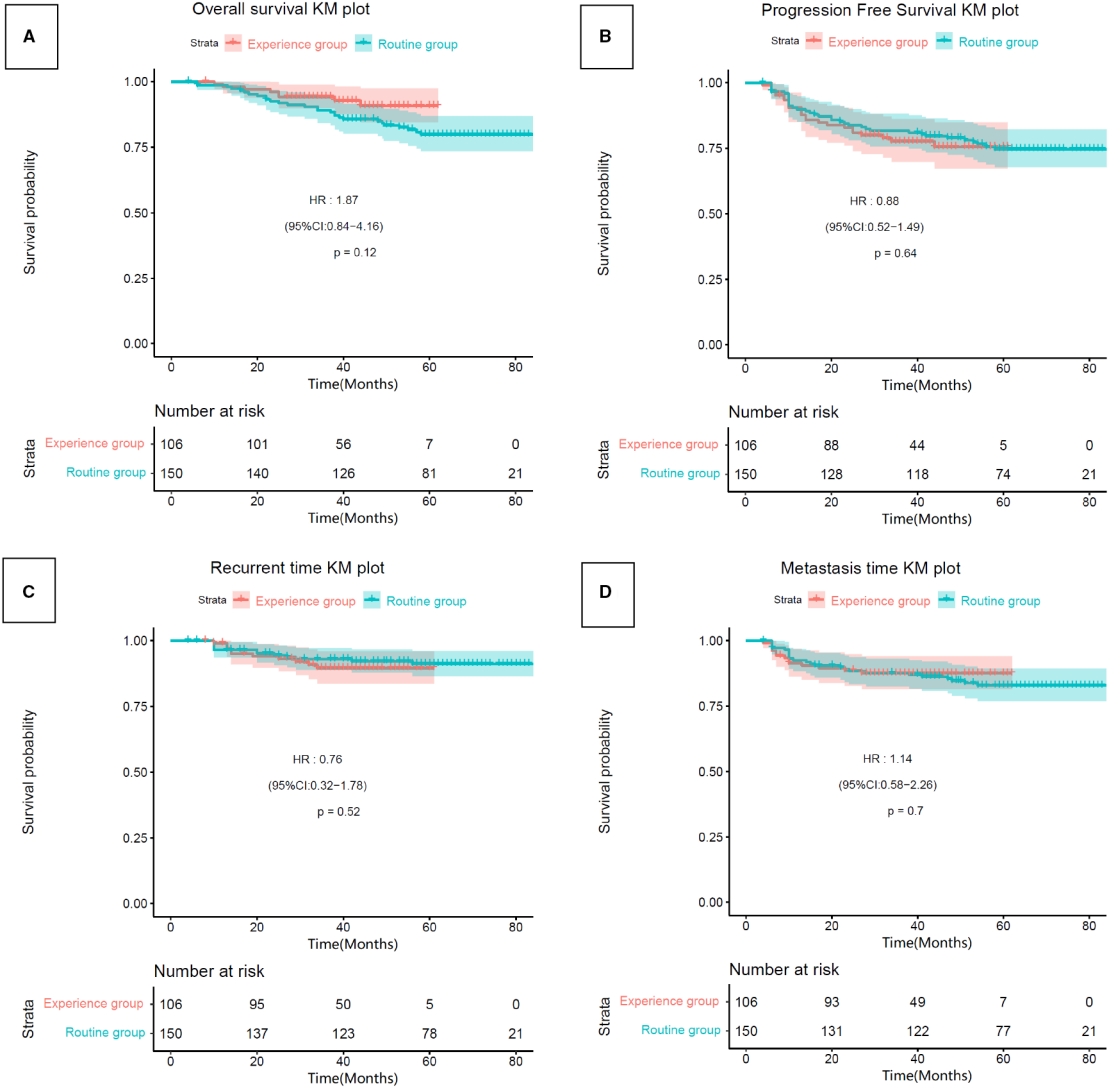
Kaplan–Meier survival curves of 256 investigated patients in the whole cohort. (A) Overall survival, (B) progression‐free survival, (C) local recurrence‐free survival, (D) distant metastasis‐free survival.

**FIGURE 2:**
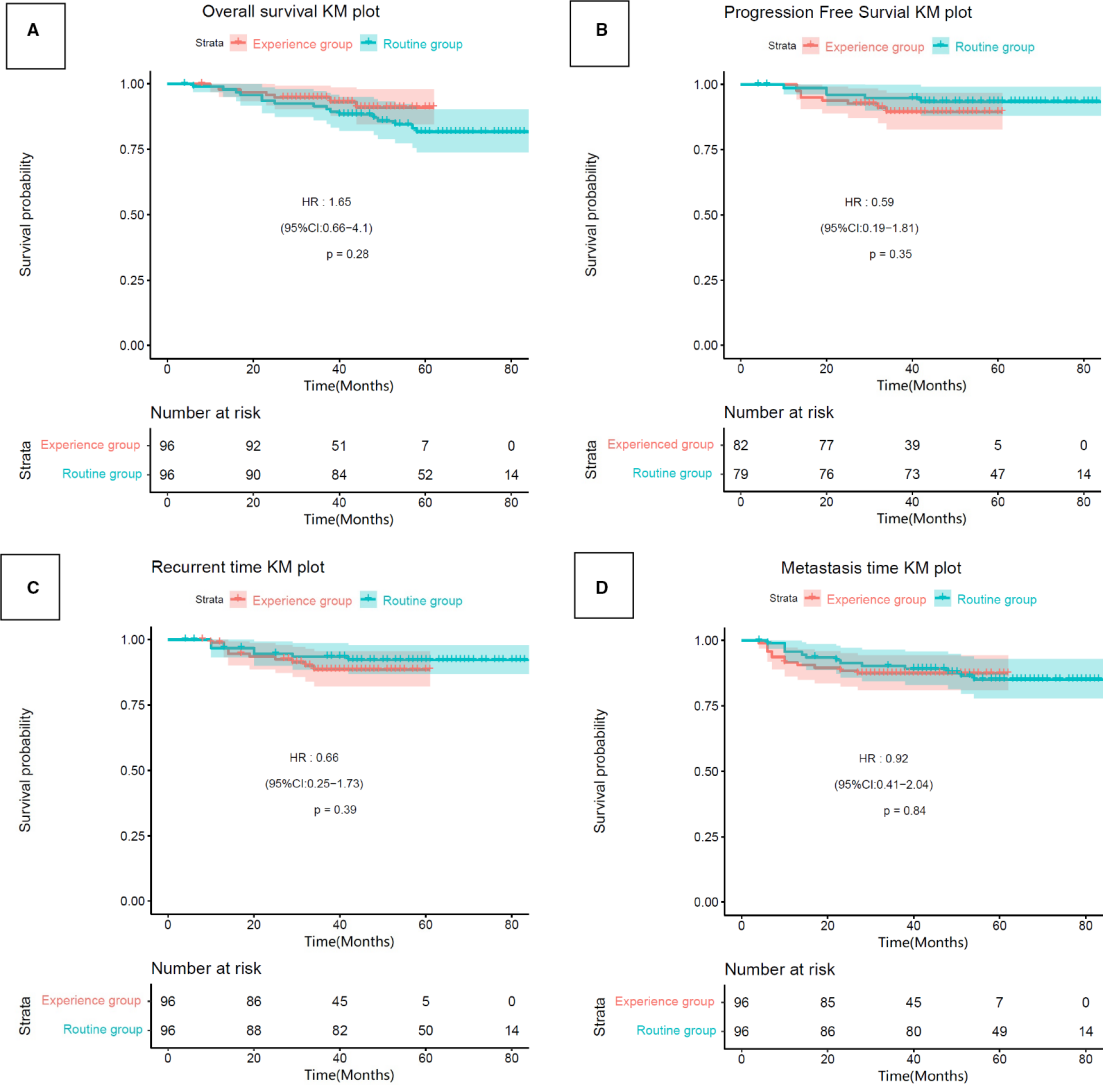
Kaplan–Meier survival curves for 192 patients in the propensity score‐matched cohort. (A) Overall survival, (B) progression‐free survival, (C) local recurrence‐free survival, (D) distant metastasis‐free survival.

For the whole cohort, the 3‐year LRFS, DMFS, OS, and DFS rates for the routine and new treatment cohorts were (93% vs. 90%, *p* = 0.532), (88% vs. 88%, *p* = 0.687), (89% vs. 94%, *p* = 0.126), and (86% vs. 85%, *p* = 0.140), respectively. For the matched cohorts, the 3‐year LRFS, DMFS, OS, and DFS rates for the routine and new treatment cohorts were (94% vs. 89%, *p* = 0.359), (93% vs. 87%, *p* = 0.487), (93% vs. 95%, *p* = 0.507), and (85% vs. 78%, *p* = 0.275), respectively. The survival curves in Figures 1 and 2 show that the 3‐year LRFS, DMFS, OS, and DFS rates were not significantly different between the groups.

### 3.3 Survival‐related independent prognostic factors

Data analysis was performed for 41 variables, including sex, pretreatment cfEBV DNA load, age, BMI, T and N categories, white blood cells, lactate dehydrogenase (LDH), the proportion of neutrophil lymphocytes, C‐reactive protein, and total treatment time, the intensity of chemotherapy, tumor regression, and adverse events. In the univariate analysis, BMI index (≥18.5 < 23.9 and ≥23.9 < 27), hemoglobin states (≥110 < 150 and >150 g/L), LDH > 245 (μ/L), pretreatment cfEBV‐DNA ≥4000, and cervical lymph nodes obtained partial remission after IC were associated significantly with failure‐free survival (FFS). Adjustment for various prognostic factors was achieved by Cox multivariate analysis of FFS including the five significant factors from the univariate analysis. The above indicators were ultimately identified as independent prognostic makers for FFS. A poorer FFS was associated significantly with a low BMI index, elevated LDH, Pre‐DNA ≥ 4000, and poor regression of cervical lymph nodes after IC chemotherapy (Table 2). Tables 2 and 3 summarize the OS, RFS, and DMFS‐related univariate and multivariate analyses.

**TABLE 2:** Univariate and multivariate analyses of prognostic factors for the whole cohort

Endpoint	Variable	Univariate analysis			Multivariate analysis		
		*p*‐value	HR	95% CI	*p*‐value	HR	95% CI
OS	BMI ≥ 18.5 < 23.9 (kg/m^2^)	0.007	0.310	0.132–0.725	0.020	0.217	0.081–0.584
	BMI ≥ 23.9 < 27 (kg/m^2^)	0.028	0.343	0.132–0.892	0.014	0.243	0.079–0.748
	HB ≥ 110 < 150 (g/L)	0.008	0.237	0.082–0.684	0.676		
	HB > 150 (g/L)	0.002	0.149	0.044–0.510	0.542		
	Pre‐EBV‐DNA > 4000 (copies/ml)	0.062	2.382	0.956–5.935	0.055	2.635	0.981–7.080
	TTT > 115 day	0.073	1.914	0.940–3.896	0.144		
	GAP > 68 day	0.037	2.133	1.048–4.341	0.014	2.874	1.233–6.697
	Responds ND PR	0.067	0.413	0.160–1.065	0.020	2.614	1.167–5.857
DFS	BMI ≥ 18.5 < 23.9 (kg/m^2^)	0.002	0.348	0.176–0.685	0.002	0.312	0.147–0.661
	BMI ≥ 23.9 < 27 (kg/m^2^)	0.016	0.399	0.188–0.844	0.011	0.339	0.146–0.784
	HB ≥ 110 < 150 (g/L)	0.009	0.290	0.114–0.736	0.747		
	HB > 150 (g/L)	0.005	0.236	0.086–0.650	0.431		
	LDH > 245 (μ/L)	0.005	2.359	1.290–4.409	0.064	0.512	0.253–1.038
	Pre‐EBV‐DNA > 4000 (copies/ml)	0.009	2.456	1.247–4.837	0.032	2.104	1.065–4.155
	Responds ND PR	0.048	1.700	1.005–2.875	0.042	1.885	1.022–3.477
DMFS	BMI ≥ 18.5 < 23.9 (kg/m^2^)	0.003	0.285	0.126–0.646	0.006	0.294	0.122–0.708
	BMI ≥ 23.9 < 27 (kg/m^2^)	0.018	0.326	0.130–0.823	0.018	0.289	0.103–0.809
	HB ≥ 110 < 150 (g/L)	0.006	0.223	0.077–0.644	0.756		
	HB > 150 (g/L)	0.005	0.184	0.057–0.599	0.704		
	LDH > 245 (μ/L)	0.002	3.104	1.502–6.415	0.003	0.294	0.132–0.652
	Pre‐EBV‐DNA > 4000 (copies/ml)	0.023	2.823	1.151–6.950	0.038	2.600	1.055–6.406
	TTT	0.077	1.862	0.935–3.708	0.543		
LRFS	Pre‐EBV‐DNA > 4000 (copies/ml)	0.043	3.627	1.042–12.620	0.059	3.360	0.957–11.791

*Note*: *p*‐values were two‐sided with significance defined as a *p*‐value <0.05.

Abbreviations: BMI, body mass index; CR, complete remission; DFS, disease‐free survival; DMFS, distant metastasis‐free survival; GAP, RT initiation date—IC initiation date; HB, hemoglobin; LDH, lactate dehydrogenase; LRFS, locoregional relapse‐free survival; ND, cervical lymph nodes; NP, nasopharynx; OS, overall survival; PR, partial remission; Pre‐EBV‐DNA, before treatment Epstein–Barr virus deoxyribonucleic acid; SD, stable disease; TTT, all treatment completion date—IC initiation date.

**TABLE 3:** Chemotherapy‐ and radiotherapy‐related acute adverse events in the matched cohort

Adverse event^b^	Routine group [cases (%)]^a^		New treatment group [cases (%)]^a^		*p*‐value
	Grade 3	Grade 4	Grade 3	Grade 4	
IC leucopenia	37 (38.5)	17 (17.7)	41 (42.7)	12 (12.5)	0.574
CCRT leucopenia	12 (12.5)	0 (0.0)	8 (8.3)	0 (0.0)	0.332
IC neutropenia	25 (26.0)	35 (36.5)	19 (19.8)	41 (42.7)	0.522
CCRT neutropenia	10 (10.4)	4 (4.2)	4 (4.2)	0 (0.0)	0.026
Febile neutropenia	8 (8.3)	0 (0.0)	7 (7.3)	0 (0.0)	0.795
IC anemia	2 (2.1)	0 (0.0)	0 (0.0)	0 (0.0)	—
CCRT anemia	1 (1.0)	0 (0.0)	0 (0.0)	0 (0.0)	0.314
IC thrombocytopenia	0 (0.0)	0 (0.0)	1 (1.0)	0 (0.0)	0.319
CCRT thrombocytopenia	0 (0.0)	1 (1.0)	0 (0.0)	0 (0.0)	0.314
IC ALT elevation	0 (0.0)	0 (0.0)	0 (0.0)	0 (0.0)	—
IC GGT elevation	1 (1.0)	0 (0.0)	0 (0.0)	0 (0.0)	0.316
IC CCR elevation	0 (0.0)	0 (0.0)	0 (0.0)	0 (0.0)	—
IC diarrhea	3 (3.1)	2 (2.1)	4 (4.2)	0 (0.0)	0.340
CCRT diarrhea	2 (2.1)	0 (0.0)	0 (0.0)	0 (0.0)	0.153
Vomiting	2 (2.1)	0 (0.0)	8 (8.3)	0 (0.0)	0.053

*Note*: *p*‐values were two‐sided with significance defined as a *p*‐value <0.05.

Abbreviations: ALT, glutamic‐pyruvic transaminase; CCR, endogenous creatinine clearance; CCRT, concurrent chemoradiotherapy; GGT, gamma‐glutamyl transpeptidase; IC, induction chemotherapy.

^a^ Percentages may not add up to 100 because of rounding.

^b^ All adverse events was based on CTCAE 5.0.

### 3.4 Adverse events

Table 3 summarizes the acute adverse events in the matched cohort related to chemotherapy. Except for 3 to 4 degrees of neutrophil toxicity during concurrent chemotherapy, there were no differences in grade 3–4 toxicity between the different treatment methods. All the acute adverse events were treated successfully using comprehensive care. In both groups, we recorded no treatment‐associated deaths.

### 3.5 Correlation analysis of the number of courses of IC

The variables studied included T and N categories, pretreatment cfEBV‐DNA load (according to the literature, 4 × 10^3^ copies/ml was determined as the pre‐DNA cutoff value), AJCC status, the intensity of IC and concurrent CT (one vs. doublet vs. triplet vs. quadruple IC, CCD < 160 mg/m^2^ vs. ≥160 mg/m^2^), total treatment time, and the GAP between the beginning of IC and CCRT, routine blood and biochemical tests, the detailed tumor invasion site of each patient for 134 anatomical structures, and treatment failure status. We observed that TPF cycles correlated positively with N stage, pretreatment cfEBV DNA load, skull base, parapharyngeal invasion, and cervical region 2 lymph metastasis (*p* = 0.034, *p* = 0.001, *p* < 0.001, *p* = 0.021, and *p* < 0.001 respectively). However, there was no correlation with post‐IC nasopharynx and neck regression response (*p* = 0.729 and *p* = 0.408, respectively). Pretreatment cfEBV DNA load correlated positively with N stage and AJCC stage (*p* = 0.012 and *p* = 0.031, respectively).

## 4 DISCUSSION

Accumulating evidence supports the use of IC before CCRT in patients with LA‐NPC; therefore, clinicians are paying more attention to the differentiation strength of IC.^29^, ^30^, ^31^ This study aimed to determine whether post ICT‐DNA could guide a risk‐adapted treatment strategy prior to CCRT implementation, to further reduce the therapeutic toxicity and improve outcomes. Based on our results, the new method did not alter the curative effect, but decreased serious neutropenia toxicity by 10% in CCRT compared with the routine strategy (hazard ration [HR] for LRFS 0.66, 95% confidence interval [CI]: 0.25–1.73, *p* = 0.39; HR for DMFS 0.92, 95% CI: 0.41–2.04, *p* = 0.84; HR for PFS 0.59, 95% CI: 0.19–1.81, *p* = 0.35). Meanwhile, the cycles of IC were reduced by 28% and the overall treatment time was shortened to a certain degree.

EBV DNA is verified biomarker for nasopharyngeal carcinoma, demonstrating clinical utility for disease surveillance, risk stratification, and population screening.^32^, ^33^ Pretreatment EBV DNA ≥2000 copies/ml was used to discriminate the IC beneficiaries in the T3N1 subgroup from among T3N0, T3N1, and T4N0 NPC cases.^34^ Guo rui proposed that stage groupings incorporating EBV DNA provided better sample size balance, outcome prediction, hazard discrimination, and hazard consistency compared with the eighth edition of the TNM staging system.^35^

Analysis of the trend of longitudinal cfEBV DNA over the IC and CCRT treatment phases allowed Lv et al. to defined four cfEBV DNA response phenotypes according to their sensitivity to treatment (early, intermediate, and late responders, and treatment‐resistant).^26^ Importantly, these phenotypic clusters were related to varying risks of tumor relapse, especially the recurrence of distant metastasis. Previous studies have shown that a high level post ICT‐DNA is an adverse prognostic factor. Among the patients who were insensitive to IC, a higher risk of locoregional relapse was mainly responsible for their survival disadvantage.^23^ Moreover, undetectable EBV DNA at mid‐treatment and post‐treatment correlated with better DMFS than did those with a detectable EBV DNA level.^36^

In Lv′s research, a biological CR (cBR) was achieved by 63.2% of patients in the IC phase; by 36.4% of patients after one cycle of IC; by 20.7% of patients after the second cycle of IC, and by 6.1% of patients post‐IC3–4. The clusters demonstrated significantly different clinical endpoints between the cBR post‐IC1 group and the cBR post‐IC2–4 group (3‐year DFS = 94.3% vs. 78.0% *p* < 0.010). The proportions of patients who received one cycle, two cycles, and three cycles of IC in the new treatment cohorts of our study were 27.%, 44.3%, and 22.6%, respectively. The results of the two research schemes are not consistent, because Lv′s study included a variety of schemes of IC, such as triplet TPF IC, docetaxel–cisplatin (TP), cisplatin–fluorouracil (PF), and gemcitabine–cisplatin (GP). Our study further confirmed that for patients showing complete regression of cfEBV DNA after IC, de‐intensification of IC might be advisable considering this subgroup's superior survival and the reduced toxicity. Beyond our clinical studies, to investigate the potential of post‐treatment EBV DNA as a biomarker to guide the further use of adjuvant chemotherapy in patients with LA‐NPC, the NRG Oncology cooperative group of the National Clinical Trial Network (NCTN) has initiated the NRG‐HN001 clinical trial (NCT02135042).^37^ In the trial, patients with undetectable post‐treatment plasma EBV DNA will be randomized to either observation or standard adjuvant chemotherapy (cisplatin and 5‐FU) after CCRT. Patients with detectable EBV DNA levels will be randomized to the standard regimen of adjuvant chemotherapy (cisplatin and 5‐FU) or the gemcitabine and paclitaxel regimen. This project is in the process of recruiting patients, and the data have not been released yet. Besides it is possible benefit to guide adjuvant chemotherapy use in patients with LA‐NPC, pretreatment EBV DNA has been assessed for its utility in guiding the use of diagnostic PET‐CT imaging^38^ and to fine‐tune prognostic tumor staging.^39^

Peng et al. found that patients with NPC with the N2–3 category who received two cycles of IC had better OS than those received 3–4 cycles (92.4% vs. 80.8%, *p* = 0.029).^40^ Similarly He et al. reported that patients receiving two to three IC cycles had similar survival, while those receiving four cycles experienced reduced survival and increased treatment‐related toxicity^41^ These results implied that patients with NPC receiving higher numbers of IC cycles might miss the optimal window of opportunity for RT, with consequent survival disadvantages. Contrastingly, Wei et al. observed that patients with N2–3 NPC receiving four cycles of IC had improved survival.^42^ The authors believed that high strength IC would reduce distant metastasis risk and increase the survival of patients at high risk. Based on TNM stage, our study incorporated post ICT‐EBV DNA as a biochemical marker which reflected the first response to IC and tumor load, which might markedly influence choices regarding the total number of IC cycles for patients with NPC. Similar studies are rare and we hope to provide evidence explaining the contradictory results.

In a 2016 study^10^ the 3 and 5 year OS and PFS rates were reported for 241 patients suffering from T3–4N1/N2–3M0 (54% stage III, 46% stage IV) NPC who were treated with TPF followed by CCRT every 3 weeks. Neutropenia (42%) and leucopenia (41%) were the most frequent grade 3 or 4 adverse events in that study. Herein, the new treatment group showed similar PFS and OS rates to those quoted in^10^: 80% and 90.0%. The incidence of grade 3–4 toxicities was similar to patients treated with the new treatment method in our research.

Some limitations ought to be highlighted. In the present study, we attempted to avoid treatment heterogeneity resulting from physician bias: The cohort comprised patients treated at a single institution by a team under a single attending doctor. Next, during CCRT, there were inconsistencies in the timing and frequency of the assessment of tumor markers. Finally, although EBV DNA PCR‐based assays are known to be vulnerable to marked inter‐laboratory variations, our cohort were treated at a single institution and thus the EBV DNA measurements may not have been affected by inter‐laboratory variation.

In summary, we investigated the effect of de‐intensifying chemotherapy in favorable LA‐NPC. We identified that more courses of IC in complete responder patients might not be effective to improve prognosis. Combining TNM stage and EBV‐DNA load after IC to determine the courses of IC in patients with LA‐NPC did not alter the curative effect, but decreased neutropenia toxicity during CCRT. We will identify these treatment‐resistant subgroups to investigate the role of chemotherapy intensification in future research.

## AUTHOR CONTRIBUTIONS

Study concepts: Qun Zhang, Zhen‐Wei Peng, Zhuo‐Sheng Gu, Wei Luo, and Yong‐Yu Mei. Study design: Wei Luo and Yong‐Yu Mei. Acquisition of data: Qun Zhang and Zhuo‐Sheng Gu. Data analysis and interpretation: Zhen‐Wei Peng, Yan Wang, Fang‐He, and Wen‐Bing Zhao. Drafting and revising the article: Qun Zhang, Zhen‐Wei Peng, and Zhuo‐Sheng Gu. Reviewing the article: Wei Luo and Yong‐Yu Mei. All authors gave final approval of the version to be published. All authors have agreed on the journal to which the article has been submitted and agree to be accountable for all aspects of the work.

## FUNDING INFORMATION

This work was supported by a grant from the Medical Science and Technology Research Projects of Health Commission of Guangdong Province [grant number A2020606].

## CONFLICT OF INTEREST

No conflicts declared.

## ETHICS STATEMENT

This study was approved by the Research Ethics Committee of Sun Yat‐sen University Cancer Center. Written informed consent was obtained from each subject before recruitment.

## CONSENT FOR PUBLICATION

All authors have read and approved the final version to be published and signed the author disclosure form.

## Data Availability

The datasets used and or analyzed during the current study are available from the corresponding author upon reasonable request.
